# When to start renal replacement therapy in critically ill patients with acute kidney injury: comment on AKIKI and ELAIN

**DOI:** 10.1186/s13054-016-1424-0

**Published:** 2016-08-06

**Authors:** Sean M. Bagshaw, François Lamontagne, Michael Joannidis, Ron Wald

**Affiliations:** 1 Department of Critical Care Medicine, Faculty of Medicine and Dentistry, University of Alberta, 2-124E Clinical Sciences Building 8440-112 ST, NW Edmonton, T6G 2B7 Canada; 2 Centre de Recherche du CHU de Sherbrooke, Sherbrooke, QC Canada; 3 Centre Hospitalier Universitaire de Sherbrooke, 3001 12e avenue N., Sherbrooke, QC J1H 5N4 Canada; 4 Division of Intensive Care and Emergency Medicine, Department of Internal Medicine, Medical University Innsbruck, Anichstrasse 35, 6020 Innsbruck, Austria; 5 Division of Nephrology, St. Michaels Hospital, University of Toronto, 30 Bond St, Toronto, Ontario M5B 1W8 Canada; 6 Department of Medicine, Université de Sherbrooke, Sherbrooke, QC Canada

**Keywords:** Acute kidney injury, Renal replacement therapy, Dialysis, Early, Delayed, Mortality, Randomized trial

## Abstract

The dilemma of whether and when to start renal replacement therapy among critically ill patients with acute kidney injury in the absence of conventional indications has long been a vexing challenge for clinicians. The lack of high-quality evidence has undoubtedly contributed decisional uncertainty and unnecessary practice variation. Recently, two randomized trials (ELAIN and AKIKI) reported specifically on the issue of the timing of initiation of renal replacement therapy in critically ill patients with acute kidney injury. In this commentary, their fundamental differences in trial design, sample size, and widely discrepant findings are considered in context. While both trials are important contributions towards informing practice on this issue, additional evidence from large multicenter randomized trials is needed.

The dilemma of when to start renal replacement therapy (RRT) among critically ill patients with acute kidney injury (AKI) when “conventional” indications are absent has long been a vexing challenge for clinicians [1, 2]. The lack of high-quality evidence has undoubtedly contributed decisional uncertainty and unnecessary practice variation [3]. Two new randomized trials focused on the timing of RRT initiation in critically ill patients with AKI have been reported recently [1, 2] (Table 1).

**Table 1:** Summary of recently published and ongoing randomized clinical trials evaluating optimal timing of initiation of RRT in ICU settings

Feature	STARRT-AKI (pilot) [6]	ELAIN [2]	AKIKI [1]	IDEAL-ICU [7]	STARRT-AKI (main)
Country	Canada	Germany	France	France	Multiple
Number of sites	12	1	31	24	>60
Number of participants	100	231	620	864^a^	2866^a^
Setting/population	Mixed medical/surgical ICU	Mixed medical/surgical ICU (94.8 % surgical)	Mixed medical/surgical ICU (79.7 % medical)	Mixed medical/surgical ICU (septic shock)	Mixed medical/surgical ICU
ARR for sample size calculation	N/A	18 %	15 %	10 %	6 %
Control group mortality	N/A	55 %	55 %	55 %	40 %
Interventions					
Early	Two of: (i) 2× increase in SCr from baseline; (ii) UOP < 6 ml/kg in preceding 12 hours; (iii) blood NGAL ≥ 400 ng/ml (within 12 hours)	KDIGO stage 2 (within 8 hours)	KDIGO stage 3 (within 6 hours)	KDIGO stage 3^b^ (within 12 hours)	KDIGO stage 2 (within 12 hours)
Delayed (conservative)	Specific criteria/emergent indications (beyond 12 hours)	KDIGO stage 3 (within 12 hours)	Specific criteria/emergent indications	Specific criteria 48–60 hours after eligibility or emergent indications	Specific criteria/emergent indications (beyond 12 hours)
Time difference	41.6 hours^c^	25.5 hours	57.0 hours	N/A	N/A
Received RRT in delayed intervention	75.0 %	90.8 %	51.0 %	N/A	N/A
RRT modality	Physician discretion	CRRT	Physician discretion (initial IHD 55 %)	Physician discretion	Physician discretion
Sepsis (%)	56 %	N/A	67 %	N/A	N/A
SOFA score of enrolled patients	~13.0	~16.0	~10.9	N/A	N/A
Mechanical ventilation (%)	93 %	88 %	87 %	N/A	N/A
Vasopressors (%)	85 %	88 %	85 %	N/A	N/A
Primary endpoint	90-day mortality	90-day mortality	60-day mortality	90-day mortality	90-day mortality
Early	38 %	39.3 %	48.5 %	N/A	N/A
Delayed	37 %	54.7 %	49.7 %	N/A	N/A

^a^Planned enrolment

^b^IDEAL-ICU protocol utilizes the RIFLE classification for AKI. RIFLE-F generally aligns with KDIGO stage 3

*ARR* absolute risk reduction, *RRT* renal replacement therapy, *SOFA* Sequential Organ Failure Assessment, *KDIGO* Kidney Disease: Improving Global Outcomes, *IHD* intermitted hemodialysis, *N/A* = not available, *NGAL* neutrophil gelatinase-associated lipocalin, *SCr* serum creatinine, *UOP* urine output, *CRRT* continuous renal replacement therapy, *RIFLE* Risk, Injury, Failure, Loss, End-Stage Kidney Disease

^c^mean hours

The Early Versus Late Initiation of Renal Replacement Therapy In Critically Ill Patients With Acute Kidney Injury (ELAIN) trial was a single-center trial comparing early RRT (starting within <8 hours of fulfilling Kidney Disease: Improving Global Outcomes (KDIGO) stage 2 AKI) with delayed RRT (starting within <12 hours of developing KDIGO stage 3 AKI or upon an absolute indication) [2]. Eligible patients were required to have blood neutrophil gelatinase-associated lipocalin (NGAL) > 150 ng/ml and at least one of sepsis, fluid overload, worsening Sequential Organ Failure Assessment (SOFA) score, or receiving vasoactive support. The trial ELAIN randomized 231 predominantly postsurgical patients. The median difference among those receiving RRT was 21 hours. Early RRT resulted in a 15.4 % reduction in 90-day mortality compared with delayed RRT (39.3 % vs 53.6 %; *p* = 0.03). Early RRT also translated into greater kidney recovery (53.6 % vs 38.7 %, *p* = 0.02; not significant after excluding deaths through 90 days), decreased RRT duration (9 vs 25 days, *p* = 0.04), shorter hospital stay (51 vs 82 days, *p* < 0.001), and reduction in selected plasma proinflammatory mediators. There were no differences in organ dysfunction scores, ICU stay, or dialysis dependence beyond 90 days.

The Artificial Kidney Initiation in Kidney Injury (AKIKI) trial was a multicenter trial that compared two strategies for starting RRT in 620 mixed critically ill patients with AKI who were receiving mechanical ventilation and/or vasoactives [1]. The early strategy started RRT within <6 hours of fulfilling KDIGO stage 3 AKI and the delayed strategy started upon fulfilling clinical criteria related to worsening AKI or complications (e.g., oligo-anuria for >72 hours; elevated urea; hyperkalemia; metabolic acidosis; and/or pulmonary edema from fluid overload). No difference in 60-day mortality was found (48.5 % vs 49.7 %, *p* = 0.79). RRT utilization differed significantly, with only 51 % of patients in the delayed strategy receiving RRT compared with 98 % in the early strategy. The median difference for starting RRT was 57 hours among those receiving RRT. In the delayed strategy, RRT-free days were greater (19 vs 17 days, *p* < 0.001) and the occurrence of catheter-related bloodstream infection (CRBSI) was lower (5 % vs 10 %, *p* = 0.03), compared with the early strategy. There was no difference in secondary endpoints including ventilator and vasoactive-free days through day 28, ICU stay, hospital stay, and 60-day dialysis.

The ELAIN and AKIKI trials are important contributions towards informing practice on this issue; however, their discordant findings necessitate careful interpretation.

Both trials were relatively small and as a result susceptible to imprecision in effect and/or limited statistical power to detect clinically important differences in survival that may result from different strategies for starting RRT [4]. The ELAIN trial was powered to detect an 18 % absolute reduction in mortality in favor of early RRT. This is an implausibly large treatment effect for any intervention in an ICU setting. This is further supported by a low Fragility Index of 3 (i.e., three more deaths in the early group or three fewer deaths in the delayed group would render the trial nonsignificant) [5]. Alternatively, the AKIKI trial was powered to show 15 % absolute reduction in mortality in favor of the delayed strategy. While conceivable that a delayed strategy may translate into fewer RRT-related complications, such an expected survival difference also seems improbable.

It is debatable whether the triggers for starting RRT in both trials reflect customary decision-making in the ICU. The criteria used for starting RRT in the early group of both trials and the delayed group in the ELAIN trial were largely based on achieving creatinine and/or urine output thresholds consistent with the KDIGO classification scheme. All participants in the early arm of the ELAIN trial commenced RRT after stage 2 AKI and the majority (91 %) in the delayed group started RRT, most often upon meeting stage 3 AKI criteria. An important consideration for clinicians is whether the triggers used for starting RRT in these trials are in fact translatable to routine bedside practice.

This issue was highlighted in the AKIKI trial, where the threshold for trial enrollment, and hence receipt of RRT in the early arm, was stage 3 AKI. During follow-up, approximately half the patients in the delayed arm did not receive RRT based on pre-established triggers. From this, one may infer that a similar proportion in the early strategy received RRT unnecessarily, and would have recovered had they not been allocated to the early arm. These observations highlight two key challenges for studies of RRT timing for AKI. In the absence of objective markers to inform a future “need” for RRT, any trial testing an early strategy of RRT initiation will inevitably enroll some patients who might never worsen to require RRT in a clinical environment where a delayed or “indication-based” approach is the standard of care. Although some would worry about the exposure of possibly unnecessary therapy, it should be emphasized that abundant data have shown that “pre-emptive” RRT is prevalent in usual practice. Moreover, neither trial showed that early RRT was incrementally harmful; although the AKIKI trial did show a modest increase in CRBSI. These points notwithstanding, we believe that the decision to start RRT in routine practice is often based on a clinical impression shaped by the patient’s global condition and trajectory, rather than thresholds of creatinine or urine output alone. Integrating the clinician’s impression regarding the likelihood of a patient needing RRT might possibly have increased the number of patients who recovered kidney function without having received RRT. More importantly, adoption of such an approach would be more consistent with the reality of clinical care which trials should strive to emulate.

The ELAIN and AKIKI trials focused attention on a controversial issue with a noteworthy evidence care gap and susceptibility to wide practice variation. However, due to fundamental differences in trial design, a relatively small sample size, and widely discrepant findings, these studies are far from definitive. Accordingly, additional evidence from large multicenter randomized trials is needed.

## Abbreviations

AKI, acute kidney injury; CRBSI, catheter-related bloodstream infection; KDIGO, Kidney Disease: Improving Global Outcomes; NGAL, neutrophil gelatinase-associated lipocalin; RRT, renal replacement therapy; SOFA, Sequential Organ Failure Assessment
